# Co-Microencapsulation of Anthocyanins from Black Currant Extract and Lactic Acid Bacteria in Biopolymeric Matrices

**DOI:** 10.3390/molecules25071700

**Published:** 2020-04-08

**Authors:** Iuliana Maria Enache, Aida Mihaela Vasile, Elena Enachi, Vasilica Barbu, Nicoleta Stănciuc, Camelia Vizireanu

**Affiliations:** Faculty of Food Science and Engineering, Dunărea de Jos University of Galati, Romania, 111, Domnească Street, 800201 Galati, Romania; enacheiulianamaria@gmail.com (I.M.E.); aida.vasile@ugal.ro (A.M.V.); elena.ionita@ugal.ro (E.E.); vasilica.barbu@ugal.ro (V.B.); nsava@ugal.ro (N.S.)

**Keywords:** black currant, anthocyanins, lactic acid bacteria, microencapsulation

## Abstract

Anthocyanins from black currant extract and lactic acid bacteria were co-microencapsulated using a gastro-intestinal-resistant biocomposite of whey protein isolate, inulin, and chitosan, with an encapsulation efficiency of 95.46% ± 1.30% and 87.38% ± 0.48%, respectively. The applied freeze-drying allowed a dark purple stable powder to be obtained, with a satisfactory content of phytochemicals and 11 log colony forming units (CFU)/g dry weight of powder (DW). Confocal laser microscopy displayed a complex system, with several large formations and smaller aggregates inside, consisting of biologically active compounds, lactic acid bacteria cells, and biopolymers. The powder showed good storage stability, with no significant changes in phytochemicals and viable cells over 3 months. An antioxidant activity of 63.64 ± 0.75 mMol Trolox/g DW and an inhibitory effect on α-amylase and α-glucosidase of 87.10% ± 2.08% and 36.96% ± 3.98%, respectively, highlighted the potential biological activities of the co-microencapsulated powder. Significantly, the in vitro digestibility profile showed remarkable protection in the gastric environment, with controlled release in the intestinal simulated environment. The powder was tested by addition into a complex food matrix (yogurt), and the results showed satisfactory stability of biologically active compounds when stored for 21 d at 4 °C. The obtained results confirm the important role of microencapsulation in ensuring a high degree of protection, thus allowing new approaches in developing food ingredients and nutraceuticals, with enhanced functionalities.

## 1. Introduction

Substitution of artificial dyes by their natural counterparts is a major challenge for the food industry, and anthocyanins have gained increasing interest as red, purple, and blue natural food pigments [1]. The increased interest for natural pigments is related to the detrimental effects of azo dyes on children’s health, although there is still doubt about the study design leading to that conclusion and to the role of preservatives that may also contribute to increased levels of attention deficit hyperactivity disorder (ADHD), as reported by [2]. However, fruits and vegetables are rich sources of biologically active compounds (BAC), as they constitute one of the important sources of potential health-promoting phytochemicals, with potent immunomodulatory, antimicrobial, and anti-inflammatory actions, inhibition of low-density lipoprotein, as well as reduction of cardiovascular diseases [3].

Black currant (*Ribes nigrum*) is a rich source of BAC such as anthocyanins, carotenoids, flavonoids, myricetin, isorhamnetin, quercetin, phenolic acids, proanthocyanidins, and vitamin C [4,5]. The amount of BAC from *Ribes nigrum* can be influenced by genotype, location, and year of collection [6]. Some studies provide evidence that these small and soft-fleshed berry fruits may have a significant impact on cancer prevention [7,8].

Phenolic compounds of black currants are represented by phenolic acids, flavonols, anthocyanins, and proanthocyanidins, with anthocyanins being the dominant group [8]. The predominant anthocyanins in black currant are delphinidin-3-*O*-glucoside, delphinidin-3-*O*-rutinoside, cyanidin-3-*O*-glucoside, and cyanidin-3-*O*-rutinoside. Nour et al. [3] identified and quantified nine individual anthocyanins in ethanolic extracts of black currants, such as delphinidin 3-glucoside, delphinidin 3-rutinoside, cyanidin 3-glucoside, cyanidin 3-rutinoside, petunidin 3-rutinoside, pelargonidin 3-rutinoside, peonidin 3-rutinoside, petunidin 3-(6-coumaroyl)-glucoside, and cyanidin 3-(6-coumaroyl)-glucoside, whereas the 3-*O*-glucosides and the 3-*O*-rutinosides of delphinidin and cyanidin accounted for 92%–97% of the total anthocyanin content. In another study, Slimestad and Solheim [9] reported the presence of fifteen anthocyanin structures, namely the 3-*O*-glucosides and the 3-*O*-rutinosides of pelargonidin, cyanidin, peonidin, delphinidin, petunidin, and malvidin; cyanidin 3-*O*-arabinoside, and the 3-*O*-(6″-*p*-coumaroyl) glucosides of cyanidin and delphinidin, as a function of genotype and of ripeness of the fruits.

The beneficial effect of anthocyanins correlated with their successful application in different formulae depends on stability and bioavailability. Due to their chemical structure, anthocyanins are sensitive compounds to extrinsic factors (pH, temperature, light, and oxygen) and interactions with food ingredients such as co-pigments, sugars, ascorbic acid, and enzymes on pigment stability [1].

The health benefits of probiotics, which are live microorganisms contained in the gastrointestinal tract, include the control of intestinal infection, serum cholesterol levels, improvements in lactose utilization, and anticarcinogenic activity with proven health benefits, including improvement in the immune system, reduction of gastrointestinal pain, and treatment of infectious bacteria [10].

However, including probiotics into foods raises some concern, due to the fact that the ingestion of food products containing probiotic bacteria is not efficient without the adequate protection against adverse conditions (e.g., gastric pH) [11]. Many probiotic bacteria are unable to survive under adverse temperature and acidity environments.

Therefore, both anthocyanins and probiotics should be protected, and the microencapsulation process is one of the most useful technologies by which the sensitive ingredients are packed within a coating or wall material. The wall material protects the sensitive ingredients against adverse reaction and controls release in the gastrointestinal environment. In addition, microencapsulation can convert liquids into powders, which are easy to handle [12]. A key element in the encapsulation process is the selection of the wall materials, allowing efficient results to be obtained, with minimal costs. Additionally, the wall materials used for encapsulation must be food-grade, biodegradable, and able to form a barrier between the internal phase and its surroundings [13]. In this regard, proteins are interesting wall materials due to their high safeness, cheapness, and food-grade variegation [14]. Among proteins, whey proteins are a favorable material for the encapsulation of bioactives, including probiotics, due to well-known properties, such as low cost and high nutritional value [15]. Whey proteins may be used alone or in combination with different polysaccharides, such as inulin and chitosan. The unique characteristics of polysaccharides for microencapsulation refer to their high modifiability, solubility, and binding ability via their functional groups [16]. Inulin, widely used as a prebiotic, is an oligosaccharide of the fructan type, found in a wide variety of plants. The selection of inulin as an encapsulating material is based on the fact that this polysaccharide cannot be digested by the enzymes of the gastric tract and has the ability to selectively stimulate the growth of beneficial bacteria of the colon [17]. Chitosan, a chitin derivative biopolymer comprising *N*-cetyl-d-glucosamine units jointed by β(1,4)-glycosidic linkages, has some exclusive characteristics due to the presence of positive-charge amino acids creating the water-soluble cationic biopolymer, with multiple applications, such as high mucoadhesive and adsorption activity, antifungal capacity, film-forming, metabolic functions, and forming micro/nanostructures [18].

Several studies have dealt with the microencapsulation of probiotics by using different combinations of biopolymers, such as whey protein and water-soluble chitosan for probiotic *Kluyveromyces marxianus* VM004 [19], symbiotic microencapsulation of *Lactococcus lactis* subsp. *lactis* R7 using whey and inulin [20], *Bifidobacterium animalis* subsp. *lactis* INL1 in whey proteins and dextrans conjugates [21], or *Lactobacillus plantarum* ATCC 8014 using dairy whey [22]. To the best of our knowledge, the microencapsulation of probiotic bacteria with anthocyanins from different sources in different combinations of biopolymers is limited. This approach allows exploitation of the biologically active potential of both anthocyanins and lactic bacteria, in a single formulation, with multiple applicability. Therefore, the main purpose of our study was to develop a proper protection microenvironment for both anthocyanins from the black currant fruits and *Lactobacillus casei* by freeze-drying, using a combination of biopolymers such as whey protein isolate (WPI), inulin, and chitosan from the perspectives of developing a multi-functional ingredient, allowing a controlled release of bioactives. The multifunctionality of the powder consisted of measuring some parameters such as encapsulation efficiency, both for anthocyanins and lactic bacteria, phytochemicals profile (anthocyanins, polyphenols, and flavonoids), color, antioxidant activity, and inhibitory effect against selected enzymes associated with carbohydrate metabolism, such as α-glucosidase and α-amylase. The powder was tested for phytochemicals stability and cell viability after storage at 4 °C for 3 months. Confocal laser microscopy was used to study the structure and morphology of the powder. Further, in vitro digestibility was applied to test the release of the anthocyanins from microcapsules in a simulated environment. The powder was tested as a functional ingredient by addition into a proper food matrix (yogurt) that, due to its compositional characteristics, ensures a high stability of anthocyanins and represents a favorable environment for bacteria. The phytochemicals and antioxidant activity of the added-value yogurt were evaluated during 21 d of storage at 4 °C. The obtained results highlighted the important role of the microencapsulation in protecting the bioactives as an efficient way to develop highly functional food ingredients. Additionally, the reported results are valuable as nutraceuticals, with an improved bioavailability for both anthocyanins and lactic bacteria, while enabling their controlled release and target delivery, and next-to-remarkable antioxidant and antidiabetic activities.

## 2. Results and Discussion

### 2.1. Co-Microencapsulation Efficiency

The biopolymers combinations used in our study allowed an encapsulation efficiency of anthocyanins in freeze-dried powder of 95.46% ± 1.30% and 87.38% ± 0.48% for lactic bacteria. Bakowska-Barczak and Kolodziejczyk [12] reported that the encapsulation efficiency of polyphenols from black currant juice in maltodextrin and inulin by spray-drying was about 86%. Mansour et al. [23] obtained the encapsulation efficiency of anthocyanins from red raspberry in soy protein isolate and acacia gum by freeze-drying of 93.05–98.87%, whereas higher values were reported by Aprodu et al. [24] for anthocyanins from black rice encapsulated in whey protein isolate, whey protein hydrolysate, and casein of 97.31% and 98.58%, depending on the wall material ratio. Yee et al. [25] encapsulated *Lactobacillus acidophilus* NCFM in mannitol by the co-extrusion technique and reported a higher encapsulation efficiency of 96.81%.

### 2.2. The Phytochemical Characterization of Co-Microencapsulated Powder

The co-microencapsulated powder showed TAC of 71.85 ± 2.33 mg cyanidin-3-glucoside equivalents (CGE)/g DW, whereas total flavonoids content (TFC) and total polyphenols content (TPC) showed values of 13.96 ± 0.21 and 12.36 ± 0.08 mg gallic acid equivalent (GAE)/g DW, respectively. The phytochemicals content led to an antioxidant activity of 63.64 ± 0.75 mg Trolox/g DW. Bakowska-Barczak and Kolodziejczyk [12] reported lower values for anthocyanins content in the powders resulting from microencapsulation of black currant juice with inulin and maltodextrin, ranging from 0.22 to 0.39 mg CGE/g DW, whereas TPC values showed significantly lower values of 0.76 to 0.96 mg GAE/g DW, leading to an antioxidant activity of 1.7–2.5 mg Trolox/100 g DW.

### 2.3. Stability over the Time of the Co-Microencapsulated Powder

The profile of phytochemicals in co-microencapsulated powder during storage is shown Table 1. The results revealed a satisfactory stability over the time of BAC into the co-microencapsulated powder, in good agreement with Bakowska-Barczak and Kolodziejczyk [12], who demonstrated that the microencapsulated anthocyanins from black currant fruits by spray-drying in different ratios of maltodextrin and inulin showed good stability over nine months of storage in the freezer. In our study, the selected microencapsulated matrices were able to create a stable barrier and maintain a high level of BAC and antioxidant activity. As can be seen from Table 1, after three months of storage at 4 °C, TAC showed good stability, with a slight decrease of approximately 27%, whereas a twofold decrease in TFC and TPC was found, leading to a slight decrease in antioxidant activity.

The cells viability of *L. casei 431*^®^ in co-microencapsulated powder was tested during storage at 4 °C after 90 d in order to test the protective effect of wall materials. The cells viability of the co-microencapsulated lactic bacteria with bioactive compounds from black currant extract during storage at 4 °C ranged from 8.13 to 6.35 log CFU/g after 90 d, while the viability of encapsulated *Lactobacillus acidophilus* NCFM with mannitol at 4 °C throughout the storage period for 30 d ranged from 8.62 to 6.80 log CFU/mL. The minimum value of the viability required for a product to be considered probiotic is 10^6^ CFU/mL [25]. Shinde et al. [26] microencapsulated *Lactobacillus acidophilus* by co-extrusion using sodium alginate solutions alone or in combination with a value-added apple skin polyphenol extract, showing the important role of microencapsulation on lactic bacteria in the improvement of the viability during storage at 4 °C for 50 d.

### 2.4. Confocal Laser Microscopy

**Figure 1:**
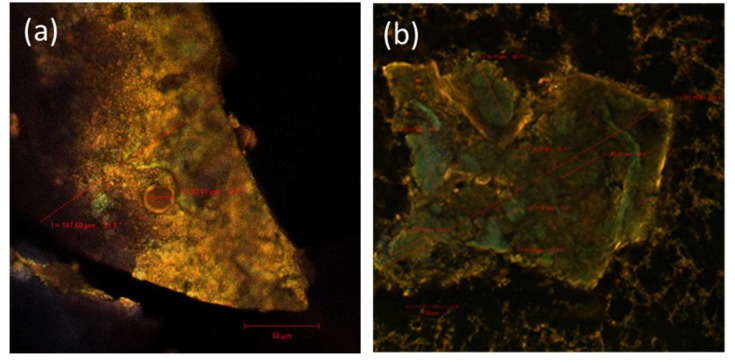
Confocal laser scanning microscopy images of the microencapsulated powder: Native state (**a**) and sample dyed with fluorophore (**b**).

It is well-known that the phenolic ring has the ability to absorb UV-visible light by displaying one intense absorption band at 280 nm (common to all phenolic substances) and another one around 520–580 nm, which is characteristic for the red anthocyanins or yellow flavonols [27]. By laser-scanning the autofluorescence of the obtained powder, large scales (147.60 µm) with irregular shape (Figure 1a) were observed. Inside them, two types of spherosomes were noticed, most of them having small diameters (under 5 µm) and also some larger ones (20.41 µm).

The phenolic compounds, due to their biochemical and molecular properties, have a wide-range emission spectrum (550–650 nm), whereas for most of the monomeric or polymeric anthocyanins, the maximum is often recorded at 585 nm [28,29].

The anthocyanins co-exist under different forms and their color intensity and tonality depend on the proportion of their different molecular structures in the plant sources [30]. For these reasons, the confocal analysis of the native microencapsulated powder revealed several isoform formations with an emission in the green-yellow domain. Through fluorescence marking with Red Congo (Figure 1b), several large coacervates of 337.60 µm were observed, coacervates that were most likely composed through the aggregation of vesicular formations (with dimensions between 15.39 and 65.11 µm) that consist of the black currants bioactives, and they are shown in green (550–580 nm). The anthocyanins aggregates were coated with the polymeric matrix, being predominantly visible in yellow. Inside and on their surface, *L. casei* 431^®^ cells were both incorporated and adsorbed so that they can be found both in the form of free clusters, inside and around the formed coacervates.

### 2.5. In Vitro Digestibility of the Black Currant Anthocyanins

**Figure 2:**
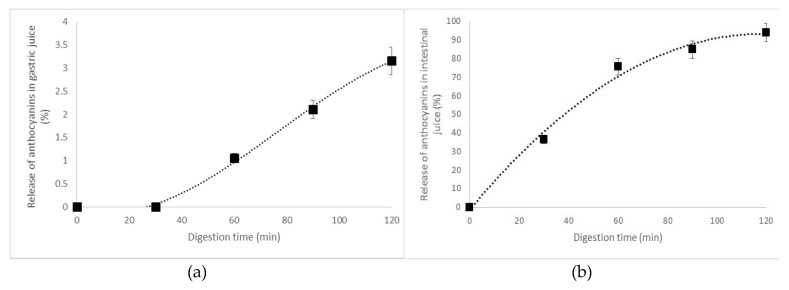
The release pattern of anthocyanins in simulated gastric environment (**a**) and controlled release in intestinal juice (**b**).

The in vitro release of anthocyanins during gastric (a) and intestinal (b) digestion is shown in Figure 2. From Figure 2a, a highly protective effect of microencapsulation on anthocyanins can be observed, with no release in the first 30 min of gastric digestion and a slow release after 90–120 min (max. 3.15% ± 0.30%).

The anthocyanins were released in the intestinal environment (Figure 2b), with a maximum release of approximately 94% after 2 h of digestion. These mean that almost the total anthocyanins were liberated from microcapsules during intestinal digestion. Our results are in good agreement with Walton et al. [31], who concluded that the major part of anthocyanins from black currant is absorbed during intestinal digestion.

### 2.6. Colorimetric Analysis

It is well known that color properties in fruits are related to pigment contents, which can be protected through microencapsulation, thus creating variations in the color parameters L*, a*, and b* [32]. The values of the chromatids and rates of color for the microencapsulated powder were L* of 30.04 ± 0.02, a* of 19.57 ± 0.09, and b* of −2280 ± 0.010. As it can be seen, the high a* value indicates a predominant reddish color of the samples, whereas the b* value suggests a lower amount of yellowness. Jimenez-Gonzalez et al. [32] encapsulated *Renealmia alpinia* (Rottb.) *Maas* fruit pericarp in maltodextrin, gum arabic, and a 1:1 mixture of both, and reported values for colorimetric parameters of 28.30 ± 1.87 for L*, 23.12 ± 3.43 for a*, and −6.58 ± 1.06 for b* for the maltodextrin:gum arabic combination. Oancea et al. [33] encapsulated sour cherry skins anthocyanins extract in whey protein isolate and reported a higher value for L* of 74.87 ± 0.29, and comparable values for a* of 19.27 ± 0.19 and b* of −2.84 ± 0.04.

### 2.7. Biological Activity

In order to demonstrate the antidiabetic and cholesterol-lowering potential, micro-encapsulated powder was tested for the inhibitory effect of enzymes involved in carbohydrate metabolism. To the best of our knowledge, no investigation has yet been carried out on freeze-dried co-microencapsulated black currant anthocyanins and lactic acid bacteria for antidiabetic potential purposes, which eventually facilitate the use of these ingredients as an anthocyanins-enriched nutraceutical. The co-microencapsulated powder showed an inhibitory effect of 87.10% ± 2.08% for α-amylase and 36.96% ± 3.98% for α-glucosidase, with a value for acarbose of 26.75% ± 1.89%. Known as oral hypoglycemic agents for diabetics, α-amylase and α-glucosidase were tested by Xu et al. [34]. These authors showed a high inhibitory effect of the polysaccharides from black currant fruits against the activity of carbohydrate hydrolyzing enzymes in the small intestine. According to this work, IC_50_ values for α-amylase for black currant and acarbose were 5.87, 3.50, 0.822, and 0.102 mg/mL, at the concentration of 4.0 mg/mL. At the same concentration, the IC_50_ values for α-glucosidase inhibition assay black currant and acarbose were 4.86, 0.697, 0.286, and 0.064 mg/mL. At a higher concentration of 4 mg/mL when compared to our study, Hussain et al. [35] encapsulated a polyherbal mixture of equal ratio of roots of *Chlorophytum borivilianum*, *Astragalus membranaceus*, *Eurycoma longifolia*, and seeds of *Hygrophila spinosa* T. Anders in 10% of gum arabic by freeze-drying and demonstrated the highest inhibition for α-amylase (93.33 ± 2.65, with an IC50 value 1.47 mg/mL ± 0.57) and α-glucosidase (73.39 ± 1.66 with an IC50 value 2.03 ± 0.45 mg/mL).

### 2.8. The Added-Value Yogurt with Powder Addition

The co-microencapsulated powder was tested as a food ingredient by addition into a yogurt in various ratios, of 2% (S1) and 5% (S2). The dairy products are particularly suitable for enrichment with co-microencapsulated anthocyanins and lactic bacteria, in terms of composition, pH, popularity with consumers, and special storage condition, later helping to maintain the stability of the added bioactives. The phytochemical parameters, in terms of TAC, TPC, and TFC, and antioxidant activity, were measured in the added-value samples after one and 21 days of storage at 4 °C (Table 2).

Increases in TAC of approximately 67% in S1 and only 12% in S2 were observed, indicating a release of anthocyanins from microparticles. TPC also increases in both samples, with approximately 34% in S1 and 26% in S2, whereas TFC decreased with only 3% in S1 and 12% in S2. As a consequence of bioactives release from the microcapsules, antioxidant activity in the added-value samples increased in S1 with 19% and slightly decreased in S2 with approximately 4%. Based on the above-mentioned results, in order to obtain a good stability of BACs over time, the powder should be added in a concentration up to 5%. However, the results suggested a satisfactory stability of the BACs in the food matrices during storage, leading to an added value when compared to the control.

## 3. Materials and Methods

### 3.1. Materials

#### 3.1.1. Chemicals and Reagents

Ethanol, methanol, 2,2-diphenyl-1-picrylhydrazyl (DPPH), 6-hydroxy-2,5,7,8-tetramethylchromane-2-carboxylic acid (Trolox), aluminum chloride, potassium acetate, Folin–Ciocalteu reagent, gallic acid, inulin from chicory, Trizma hydrochloride, pepsin from gastric porcine, pancreatin, chitosan, acetic acid, and hydrochloric acid were purchased from Sigma Aldrich Steinheim (Taufkirchen, Germany). Sodium bicarbonate was purchased from Honeywell, Fluka (Germany), whereas WPI 894 was from Fonterra (Clandeboye, New Zealand). The commercial culture *Lactobacillus casei* ssp. *paracasei* (*L. casei* 431^®^) was provided by Chr. Hansen (Hoersholm, Denmark). De Man, Rogosa and Sharpe agar (MRS agar) from Merck (Darmstadt, Germania) was used for evaluation of the viability of the *L. casei* 431^®^. *p*-Nitrophenyl-α-d-glucopyranoside, α-glucosidase from *Aspergillus niger* (≥750 U/g), and α-amylase from porcine pancreas (Type I-A, 700–1400 units/mg protein) were purchased from Sigma-Aldrich Corp. (St. Louis, MO, USA).

#### 3.1.2. Sample Processing

The black currant fruits were purchased from a local market (Galati, Romania) in October 2019. In order to obtain the aqueous extract, the fruits were washed with distillated water and dried with a paper towel. One-hundred grams of fruits were grinded and mixed with 300 mL of distillated water at 45 °C. The extract was sonicated for 30 min at 40 °C (MRC Scientific Instrument) and centrifuged at 5000× *g* for 30 min at 4 °C (cooling centrifuge Hettich Zentrifugen, Universal 320R, Tuttlingen, Germany). In the resulting aqueous extract (200 mL), 4 g of WPI (2%), 2 g of chitosan (1%), and 2 g of inulin (1%) (ratio of 2:1:1, *w*:*w*:*w*) were dissolved. The mixture was allowed to homogenize until complete hydration using an orbital shaker (orbital shaking incubator with electronic frequency control) for 4 h at 37 °C and 150 rpm. After complete hydration, the solutions were sterilized using a UV lamp and inoculated with 1 g of *L. casei 431^®^* lyophilized culture and freeze-dried (CHRIST Alpha 1–4 LD plus, Osterode am Harz, Germany) at −42 °C under a pressure of 10 Pa for 48 h. Afterward, the powder was collected and packed in metallized bags, and kept at 4 °C until further analysis.

### 3.2. Encapsulation Efficiency

The encapsulation efficiency was estimated by evaluation of surface anthocyanins content (SAC) and total anthocyanins content (TAC). For TAC evaluation, 0.2 g of co-microencapsulated powder was dissolved in 5 mL of a methanol:acetic acid:distillated water mixture, in a ratio of 25:4:21, homogenized, and sonicated to destroy the capsules. The absorbance was measured at wavelengths of 520 and 700 nm. The results are expressed as mg of cyanidin-3-glucoside equivalents per g dry weight of powder (mg CGE/g DW). To investigate SAC, the procedure involved the addition of a mixture of methanol:ethanol in a ratio of 1:1 to 0.2 g of powder, followed by the same procedure as for TAC, skipping the homogenization and sonication steps. The encapsulation efficiency was calculated as follows:

(1) $$EE\left(\%\right)=\frac{TAC-SAC}{TAC}\times 100$$

For lactic bacteria encapsulation efficiency estimation, the procedure described by Colín-Cruz et al. [11] was used. Quantification of the viable bacteria was performed by the pour plate technique. The percentage (%) of efficiency was determined according the following equation:

(2) $$EE\;\left(\%\right)=\frac{N}{N_{0}}\times 100$$

where *N* is the number of viable cells (CFU/g) in the powder and *N_0_* is the number of viable cells in the solution before the freeze-drying process.

### 3.3. The Phytochemicals Content

The powder was analyzed in terms of total monomeric anthocyanins (TAC), total polyphenols content (TPC), total flavonoids content (TFC), and antioxidant activity as described by Oancea et al. [33]. TPC was analyzed by the Folin–Ciocâlteu method and expressed as mg gallic acid equivalent (GAE)/g DW and TFC by the aluminum chloride method and expressed as mg catechin equivalents (CE)/g DW. The antioxidant activity of the co-microencapsulated powder was evaluated by estimating the DPPH free radical-scavenging activity, involving the addition of 0.1 mL of the solution resulting from powder extraction (TAC evaluation) to 3.9 mL of DPPH solution (0.1 M). After a 90 min incubation period at ambient temperature in the dark, the absorbance at 515 nm was measured. The scavenging percentage of DPPH was expressed as mMol Trolox/g DW using a calibration curve.

### 3.4. Storage Stability

The TAC, TFC, TPC, and antioxidant activity were analyzed during three months of storage at 4 °C in the dark by using the above-mentioned methods.

### 3.5. Structure and Morphology of the Microparticles

In order to observe the structure and morphology of the microparticles obtained by co-microencapsulation, an LSM 710 Carl Zeiss confocal laser scanning microscope was used. The system comprises 4 lasers, namely a diode laser (405 nm), Ar-laser (458, 488, 514 nm), DPSS laser (diode-pumped solid-state, 561 nm), and He–Ne laser (633 nm). The images were analyzed and rendered with the ZEN 2012 SP1 black edition software. To observe the fluorescence, the samples were assessed both in their native state and dyed with the Red Congo (40 μM) fluorophore.

### 3.6. In Vitro Digestibility of the Anthocyanins

The release pattern of anthocyanins was evaluated under simulated gastric juice (SGJ) and simulated intestinal juice (SIJ), as described by Oancea et al. [33]. The static system included the use of pepsin (HCl buffer pH 2.0) and pancreatin (sodium bicarbonate buffer, pH 7.8), for 2 h for each phase at 37 °C. The anthocyanins were measured every 30 min by the pH differentiation method.

### 3.7. Colorimetric Analysis of the Microencapsulated Powder Using CIEL*a*b* System

The CIE L*a*b* system is the most common tool for analyzing the color characteristics of the products [36]. It is based on a brightness index, noted with L*, two color characteristics, denoted with a* and b* [37], alongside the coordinate C* (chromatic, the value being directly proportional to chromatics) and h* (the hue value in the range 0–360°: Red to 0°, yellow to 90°, green to 180°, blue to 270°) [38]. The factor L* may have values in the range from 0 (for black) to 100 (for white). It should be considered that for the two characteristics, a* has negative values for colors closer to the green shade and positive values for colors closer to red, while b* has positive values for colors closer to yellow and negative values for blue colors [36].

### 3.8. Viability of Lactic Acid Bacteria

The viability test was done immediately after the encapsulation process was completed (day 0), after 21 d and three months at 4 °C. For viable cell counting of the *L. casei 431^®^* culture, the pour plate technique was employed. The viable cell number was determined by estimating the number of colony-forming units by cultivation on the MRS-agar plates (medium at pH 5.7) after incubation at 37 °C for 48 h. The counts were expressed as colony-forming units per g DW [39].

### 3.9. Inhibitory Activity

The powder was dissolved in sodium phosphate buffer (0.1 M, pH 6.9) at a concentration of 1 mg/mL. The α-glucosidase and α-amylase inhibitory activity of the co-microencapsulated powder was measured as described by Costamagna et al. [40].

### 3.10. Added-Value Food Products with Co-Microencapsulated Powder

The co-microencapsulated powder was added to yogurt (50 g) in a ratio of 2% (S1) and 5% (S2) and stored at 4 ± 1 °C in the absence of light for 21 d. A control sample without the addition of powder (0.50 g) was prepared as a control. The containers were withdrawn at day 0 and after 21 d to determine TAC, TFC, TPC, and antioxidant activity.

### 3.11. Statistical Analysis

Statistical analysis of data was performed using Minitab19 (Minitab Inc., State College, PA, USA). All experiments were performed in triplicate, and values were expressed as mean values ± standard deviation. The differences between samples were assessed using one-way ANOVA after testing the normality and homoscedasticity conditions. Post-hoc analysis via Tukey’s method was employed when the p-value resulting from ANOVA was lower than 0.05.

## 4. Conclusions

In this study, the black currant was selected as a potential source of bioactives, which were co-microencapsulated with lactic bacteria, using a combination of whey protein isolate, inulin, and chitosan, in order to develop a highly functional ingredient with multiple applications. The freeze-drying method allowed a dark purple powder to be obtained, with a satisfactory content of phytochemicals and antioxidant activity, whereas lactic acid bacteria were found in a number of 11 log CFU/g DW. Large shapes, including two types of spherosomes, with small and large diameters, corresponding to anthocyanins and lactic acid bacteria were observed. The anthocyanins aggregates were coated with the polymeric matrix. Inside and on their surface, *L. casei* 431^®^ cells were both incorporated and adsorbed; therefore, both bounded and free clusters, inside and around the coacervates, were observed. The powder revealed a good stability of both phytochemicals and cell viability during storage for 90 d, whereas the encapsulants showed a significant protective effect on anthocyanins during in vitro digestibility. Additionally, the co-microencapsulated powder showed a significant antidiabetic effect, by inhibiting the enzymes associated with metabolic syndrome. In order to test the powder as a functional ingredient, different ratios were added into a yogurt and the stability of phytochemicals was evaluated during 21 d at 4 °C. The results have highlighted the satisfactory stability of the powder in the food matrices, the added value being demonstrated by the high content of biologically active compounds and antioxidant activity. Due to the demonstrated properties, the obtained powder may be a successful candidate for natural pigments, replacing synthetic colorants commonly used in the food industry, thus contributing to the potential health benefits associated with the consumption of anthocyanins and lactic bacteria.

## Figures and Tables

**Table 1:** Storage stability of co-microencapsulated bioactives in powder.

Phytochemicals	0	90 Days
Anthocyanins (mg CGE/g DW)	71.85 ± 2.33 ^a^	52.99 ± 5.18 ^b^
Polyphenols (mg GAE/g DW)	12.36 ± 0.08 ^a^	6.16 ± 0.18 ^b^
Flavonoids (mg CE/g DW)	13.96 ± 0.21 ^a^	7.76 ± 0.88 ^b^
Antioxidant activity (mMol Trolox/g DW)	63.64 ± 0.75 ^a^	62.36 ± 0.03 ^b^

Mean values that, for the same line, do not share the same superscript letter are statistically significant at *p* < 0.05 based on Tukey’s method.

**Table 2:** The phytochemical profile of the added-value yogurt samples.

Phytochemicals	Storage Time (d)	Control	S1	S2
Antioxidant activity (mMol Trolox/g DW)	0	2.3 ± 0.26 ^B,c^	11.95 ± 3.45 ^A,b^	27.33 ± 3.17 ^A,a^
	21	9.44 ± 2.84 ^A,b^	14.22 ± 2.00 ^A,b^	26.24 ± 0.68 ^A,a^
Polyphenols (mg GAE/g DW)	0	3.91 ± 0.02 ^B,a^	5.32 ± 0.09 ^B,b^	6.48 ± 0.49 ^B,c^
	21	5.76 ± 0.53 ^A,b^	7.1 ± 0.46 ^A,a^	8.15 ± 0.23 ^A,a^
Flavonoids (mg CE/g DW)	0	95.63 ± 1.38 ^A,a^	99.59 ± 5.56 ^A,a^	101.50 ± 6.50 ^A,a^
	21	71.26 ± 6.23 ^B,b^	97.50 ± 2.26 ^A,a^	90.09 ± 0.72 ^B,a^
Anthocyanins (mg CGE/g DW)	0	0.80 ± 0.11 ^A,c^	11.84 ± 4.43 ^A,b^	27.75 ± 0.24 ^B,a^
	21	0.98 ± 0.52 ^A,c^	15.52 ± 0.55 ^A,b^	32.64 ± 0.42 ^A,a^

For each phytochemical tested, mean values that, for the same sample, do not share the same superscript letter (A, B) are statistically significant at *p* < 0.01 based on Tukey’s method. The mean values that, for the same storage time, do not share the same superscript letter (a, b, c) are statistically significant at *p* < 0.05 based on Tukey’s method. Control—yogurt with no added co-microencapsulated powder; S1—yogurt with 2% addition of co-microencapsulated powder; and S2—yogurt with 5% addition of co-microencapsulated powder.
